# Replacing Animal Meat with Plant-Based Meat Alternatives: The Impact of Protein Quality on Protein Adequacy in the Dutch Diet

**DOI:** 10.1016/j.cdnut.2025.104562

**Published:** 2025-02-05

**Authors:** Anne J Wanders, Samantha N Heerschop, Sander Biesbroek, Mariska Dötsch-Klerk

**Affiliations:** 1 Unilever Foods Innovation Centre, Wageningen, The Netherlands; 2 Division of Human Nutrition and Health, Wageningen University and Research, Wageningen, The Netherlands

**Keywords:** protein quality, protein adequacy, diet modeling, plant-based meat alternatives, meat replacer, protein digestibility-corrected amino acid score (PDCAAS)

## Abstract

**Background:**

A shift to more plant-based consumption patterns may lower the protein adequacy of diets.

**Objectives:**

The objective of this study was to examine how replacing animal meat with plant-based meat alternatives impacts protein adequacy in the Dutch diet by considering protein quality data.

**Methods:**

Habitual total and utilizable protein intakes were calculated from meal-based food consumption data from 1633 participants aged 18 to 70 y of the Dutch National Food Consumption Survey 2012–2016. Utilizable protein intake was calculated as the sum of protein intake per meal adjusted for protein digestibility-corrected amino acid score and compared to the estimated average requirement for Dutch adults to calculate the percentage of the population with an adequate protein intake. In the modeling scenarios, all animal meat was replaced gram-for-gram with meat alternatives from various protein sources currently available on the Dutch market.

**Results:**

Replacing all meat with meat alternatives decreased the intake of animal protein from 59% to 36%, median total protein intake from 1.14 g/kg/d to 1.09 g/kg/d, median utilizable protein intake from 0.94 g/kg/d to 0.86 g/kg/d, and protein adequacy from 93% to 86%. Additional scenarios indicated that the protein adequacy was mostly impacted by total protein content, lysine content, and protein digestibility of the meat alternatives.

**Conclusions:**

This modeling study indicated that when all animal meat was replaced by plant-based meats, total and utilizable protein intake remained adequate for the majority (86%) of the Dutch adult population. Individuals relying primarily on plant-based protein should ensure a sufficient total protein intake from a variety of sources.

## Introduction

The global production and consumption of animal meat have increased substantially over the past decades [1]. Excess consumption of red and processed meat has been associated with negative environmental and health effects [2]. Therefore, a shift toward consuming less meat is recommended [3,4]. One way to support this shift is through the use of plant-based meat alternatives (PBMAs) designed to mimic the appearance, taste, and texture of meat. People who eat meat regularly may find it easier to lower its consumption by using PBMA, as these can replace meat in their usual dishes without a need to change eating and cooking habits [5,6]. For the purpose of this work, the term PBMA is used for products of both plant and fungal (mycoprotein) origin. Fungi-based foods biologically do not belong to the plant kingdom but are used for the same purpose: to replace animal meat.

Because of several differences between plant-based and animal-based protein-rich foods, a shift to more plant-based consumption patterns may lower the protein adequacy of diets. Compared to animal-based foods, plant-based foods generally contain lower amounts of protein [7]. In addition, they usually have a less optimal indispensable amino acid (IAA) pattern compared to requirements [8], a lower protein digestibility [9], and, therefore, a lower protein quality. IAAs from individual foods can complement one another to achieve optimal amino acid patterns [8]. However, it is unclear how a lower protein quality of an individual food like PBMA impacts the utilizable protein intake from a meal or a diet, particularly in populations reliant on plant-based sources.

An earlier study in French adults that modeled the impact of increasing the plant-to-animal protein ratio on protein adequacy [10] found that protein adequacy is primarily a matter of protein quantity, not protein quality, under the condition that the consumed plant proteins came from a mixture of plant-based protein sources such as grains, legumes, nuts, and seeds. However, in that study, the modeling was done with whole food plant-based foods and did not include PBMA. An additional potential methodological limitation was that utilizable protein intake was calculated at the daily level rather than per meal. Given that there is a lack of scientific consensus on whether the human body can store amino acids and complement deficiencies in specific amino acids at breakfast with a surplus of those amino acids at dinner [11], it is recommended to calculate daily utilizable protein intake by summing utilizable protein intakes within shorter time windows, for example per meal moment [12].

People are increasingly consuming PBMAs, so it is important to study how this type of product impacts protein intake. This study aimed to examine how replacing animal meat with PBMA impacts protein adequacy in the Dutch diet by considering protein quality data and calculating utilizable protein per meal moment. Dutch dietary data were selected because these represent protein intake and sources in Western Europe [13] with a relatively high intake of PBMA [14]. In addition, meal-specific food intake data and protein quality data were available for this population [15].

## Methods

### Study population and dietary assessment

Nationally representative food consumption data on 1633 males and females aged 18–70 y from the Dutch National Food Consumption Survey conducted between 2012 and 2016 were used [16]. In this survey by the National Institute for Public Health and the Environment (RIVM), food consumption data were based on 2 nonconsecutive 24 h dietary recalls, allotted over seasons and days of the week, including week and weekend days. Trained dietitians conducted standardized recalls using the GloboDiet computer program that was developed by the International Agency for Research on Cancer [17]. Food intake was captured along with information on meal moments. A detailed description of the recruitment and data collection is given elsewhere [16].

### Food composition data

Total protein intake was calculated using the Dutch Food Composition Database (NEVO) [Online version 2016/5.0, RIVM] [7]. Intakes of amino acids and protein digestibility were calculated using a database generated by Heerschop et al. [15]. In brief, the amino acid composition was assigned per NEVO food code based on similar foods from the Danish (Frida), American (USDA), English (McCance and Widdowson), and Japanese food composition tables. Mean protein digestibility factors were assigned per food group based on literature research [15]. The sum of amino acids was standardized against total protein per NEVO food code.

### Modeling scenarios and assumptions

To examine how replacing meat with PBMA affects total diet protein intake, several scenarios were formulated, as shown in Table 1 [18,19]**.** The “Current diet” was calculated based on the actual survey data. The “No protein replacement” scenario evaluated the impact of eliminating all meat without introducing any substitutes as an extreme representation of individuals simply omitting meat from their diet without considering an alternative protein source. In the “Current PBMA” scenario, the impact of replacing all meat with PBMA, which is currently available in the Dutch market, was evaluated (details provided in the next section). To explore the impact of protein content and protein digestibility, 2 additional scenarios were formulated with current PBMA. In the “Equal protein PBMA” scenario, the total protein content of the current PBMA was increased to be similar to the protein content of meat. In the “Low digestible PBMA” scenario, the digestibility of current PBMA was lowered to 75%, which represents the mean digestibility factor of the food group legumes [15]. This digestibility factor likely underestimates the actual digestibility of PBMA because processing generally increases digestibility [9].

**TABLE 1:** Modeling scenarios.

Scenario	Protein source	Protein content, g/100 g^1^	Protein digestibility %^2^
Current diet	Animal meat	18.8	95
No protein replacement	-	-	-
Current PBMA	Current PBMA^3^	15.4	94^4^
Equal protein PBMA	Current PBMA	18.8	94^4^
Low digestible PBMA	Current PBMA	15.4	75
Protein source-specific scenarios			
10% Mycoprotein	Mycoprotein	10	78
10% Pea	Pea	10	92
10% Soy	Soy	10	95
10% Wheat	Wheat	10	95
25% Mycoprotein	Mycoprotein	25	78
25% Pea	Pea	25	92
25% Soy	Soy	25	95
25% Wheat	Wheat	25	95

Abbreviation: PBMA, plant-based meat alternatives.

1 Mean protein content of animal meat was calculated from the current diet. The mean protein content of PBMA was estimated from the data on the currently available PBMA. The 10% and 25% protein content were based on the protein range in the currently available PBMA.

2 True protein digestibility data of soy protein concentrate, pea protein concentrate, wheat meat analog, and mycoprotein were taken from [18,19]. The protein digestibility of PBMA in the low digestible PBMA scenario was standardized for all protein sources and represents the digestibility of mean whole-food legumes.

3 Protein source of current PBMA is based on the contribution of each protein source as currently used in PBMAs available on the Dutch market (Supplemental Table 1).

4 Weighted mean digestibility; see Supplemental Table 1 for digestibility factors and weighting factors for each of the protein sources.

Subsequently, 8 protein source-specific scenarios were specified to further explore the impact of varying amino acid patterns, digestibility, and protein contents in PBMA. Soy, pea, wheat, and mycoprotein were selected because of their variation in amino acid patterns and digestibility, and for each source, a 10% (10 g/100 g) and 25% (25 g/100 g) scenario was defined, representing the range of protein content in currently available PBMAs.

Modeling was done under the assumption that all consumed animal meat was replaced gram-for-gram. Other dietary intakes and food choices were left unchanged.

### PBMAs currently available on the market

The composition of PBMA currently available on the Dutch market was based on the online PBMA assortment of the Dutch supermarket Albert Heijn in June 2022. Ingredient lists of 121 PBMA that intended to mimic animal meat were extracted for protein content (g/100 g) and protein sources (percentage). The majority (96%) of ingredient lists contained information on the percentage contribution of key protein ingredients. This information was used to approximate the contribution per protein source for each product. For example, when the ingredient list mentioned soy structure (64%) and fava beans, we approximated the contribution of soy to be 64% of the protein in this product and fava beans to be 36% (See Supplemental Text 1 for a detailed example). This data was used to estimate the proportional contribution of each protein source to all PBMA available on the market. The data indicated that in currently available PBMA, 55% of the protein comes from soy, 23% from wheat, 11% from peas, 5% from milk, 4% from fava beans, and 3% from mycoprotein and that mean protein content is 15.4 g/100 g product (Supplemental Table 1).

Subsequently, in the “Current PBMA” scenarios, the proportional contribution per protein source was used to assign PBMA from the 6 protein sources to individuals. For example, in 55% of the individuals, animal meat was replaced with soy-based PBMA; in 11% of individuals, animal meat was replaced with pea-based PBMA, etc. This method assigned single protein sources and not protein blends. It should be noted that, in reality, PBMA is often a protein blend. Therefore, the current approach may potentially underestimate protein quality.

### Amino acid composition and protein digestibility of PBMAs

For the purpose of this study, we added amino acid composition and protein digestibility factors specifically for PBMA made from soy, pea, wheat, mycoprotein, milk, and fava beans to the food composition database. The amino acid composition of soy, pea, wheat, and mycoprotein was defined based on analytical data of PBMA available on the Danish market and intended for food composition databases [20]. In the Danish study, information on amino acid composition was provided per product type (mince, pieces, and sausages) and by protein source (soy, pea, wheat, and mycoprotein). To obtain data on single protein sources (excluding protein blends), we screened the ingredient lists of the samples taken per product type and protein source and selected the data of the product type with the highest rate of single protein sources. For example, we selected “soy pieces” as none of the 6 samples in this product type contained a second protein source, unlike the other product types that included blends [20]. Based on this approach, the amino acid composition of soy pieces, pea mince, seitan (wheat), and mycoprotein mince were selected to represent the PBMA in the current study. The amino acid composition of fava beans was taken from Nosworthy et al. [21] and of milk and animal meat from the available food composition database [15]. The true total protein digestibility of the “Low digestible PBMA” scenario was set at 75% for all protein sources, based on the digestibility of cooked whole-food legumes in the available food composition database [15]. True total protein digestibility of the other protein sources was based on Miller et al. [18] and the federal register [19]. Protein digestibility-corrected amino acid scores (PDCAAS) and digestibility factors are presented in Tables 1 [18,19] and 2 [15,20,21,22]. To calculate PDCAAS scores, the amino acid reference pattern for older children, adolescents, and adults was used [22]; this reference pattern was deemed most appropriate for the current study population and is the most up-to-date assessment of amino acid requirements.

**TABLE 2:** Amino acid reference pattern, protein digestibility-corrected amino acid ratios (percentage), protein digestibility-corrected amino acid scores and limiting amino acids of protein sources.

Protein sources^1^	His	Ile	Leu	Lys	Met+ Cys	Phe+ Tyr	Thr	Trp	Val	PDCAAS	Limiting AA
	Protein digestibility-corrected amino acid ratios relative to reference pattern (%)										
Mean animal meat	201	158	124	178	143	175	166	161	133	1.24	Leu
Current PBMA	145	133	117	109	108	197	140	175	110	1.08	Met+ Cys
Mycoprotein	119	126	101	131	126	163	165	201	120	1.01	Leu
Pea	144	136	120	136	73	202	137	141	114	0.73	Met+ Cys
Soy	154	142	120	128	105	201	157	199	113	1.05	Met+ Cys
Wheat	119	107	105	34	128	196	97	145	90	0.34	Lys

Reference pattern^2^	Amino acid requirement (mg/g)										

Older children, adolescents, and adults (>3 y)	16	30	61	48	23	41	25	6.6	40		

Abbreviations: AA: amino acid; Cys: cysteine; His: histidine; Ile: isoleucine; Leu: leucine; Lys: lysine; Met: methionine; PBMA: plant-based meat alternative; PDCAAS: protein digestibility-corrected amino acid score; Phe: phenylalanine; Thr: threonine; Trp: tryptophan; Tyr: tyrosin; Val: valine.

1 Amino acid composition data on soy, pea, wheat, and mycoprotein taken from [20], on fava bean (cooked) from [21], and on milk and mean weighted animal meat from [15].

2 Older child, adolescent, and adult amino acid reference pattern from [22].

### Calculation of utilizable protein intake and protein adequacy

In the present study, protein adequacy was calculated by first adjusting crude protein intake per meal for the protein quality of foods [[22], [23], [24]] and subsequently calculating whole-day protein adequacy as described in detail by Heerschop et al. [15]. In brief, this involved meeting 3 components: *1*) IAA requirements per meal, taking into account protein quality; *2*) dispensable amino acid (DAA) requirements per meal to complement IAAs; and *3*) total daily protein requirements [24]. Utilizable IAA per meal was calculated by multiplying the total amount of protein per meal by the PDCAAS per meal. This represents the proportion of each IAA in the reference pattern that can be digested and subsequently utilized for protein synthesis in the body. The appropriate amount of DAAs needed to complement IAAs in each meal was calculated using the IAA to DAA ratio 0.29:0.71 [22,24]. It was assumed that the surplus of IAAs was 100% converted into DAAs; therefore, the ratio of IAA:DAA was always met, and DAAs could not be limited. The total daily protein requirement was based on the estimated average requirement (EAR) of 0.66 g/kg body weight/d for dietary protein intake for adults [25], of which we assumed it to be 100% utilizable. The Health Council of the Netherlands proposed calculating individual protein requirements by using reference body weight instead of actual body weight to account for the increase in overweight in the population [25]. Reference body weight was calculated by squaring individual height in meters and multiplying this by a BMI (in kg/m^2^) of 22 for adults aged 18–50 and 23 for adults aged 50–70 [25]. Utilizable protein intake per meal across the day was summed and then expressed as total utilizable protein in grams per kilogram per day (g/kg/d).

Subsequently, daily total and utilizable protein intake weighted for demographics, season, and week or weekend day and corrected for the intra-individual (day-to-day) variance were calculated using Statistical Program to Assess Dietary Exposure (SPADE) [26] to provide habitual population-representative intakes. In SPADE, data are normalized, modeled as a function of age, and parameters calculated to obtain a shrunken distribution. A back transformation is then performed to return to the original scale and remove within-person variance [26]. Protein adequacy was evaluated as the percentage of the population with an adequate protein intake by dividing habitual total and utilizable protein intakes by the EAR and calculating the percentage of individuals with a ratio >1.

### Data analysis

The modeling scenarios were calculated using DaDiet Software (Dazult Ltd) [27]. DaDiet is a web-based software tool that allows accurate estimation of exposure to nutrients and substances added to foods, including contaminants, food additives, and pesticides. Modeling output containing data on total protein, digestible protein, and amino acid intakes by study day, meal occasion, and food code was transferred to R (version 4.4.0), where habitual total and utilizable protein intake were calculated as outlined above and described in detail by Heerschop et al. [15]. Additionally, total diet protein digestibility, PDCAAS per meal, and limiting amino acids per meal were calculated. Results of the habitual intake distribution were presented as median and fifth and 95th percentiles. Stratified analyses for age and sex were all performed in a similar way, assuming no differences in requirements.

## Results

**FIGURE 1:**
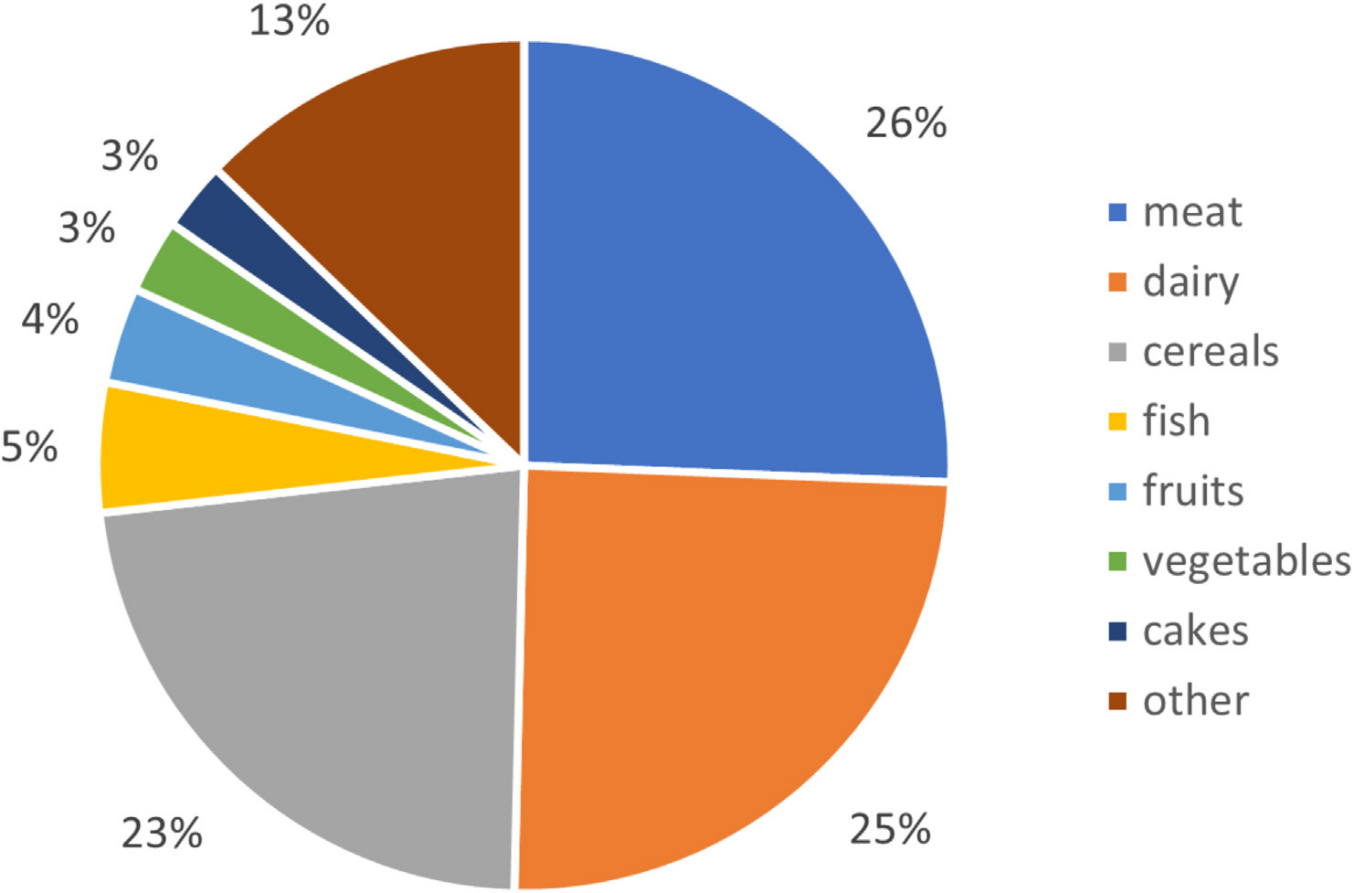
Percentage protein intake by food group in current diet.

In this population of 812 males and 821 females, median total protein intake was 1.21 (5th and 95th percentile: 0.85–1.67) g/kg/d in males and 1.07 (0.75–1.46) g/kg/d in females. The distribution of protein intake across food groups is shown in Figure 1. Some food groups include proteins from both animal and plant sources. Of the total protein intake, 59% was from animal origin. The primary sources of animal-based protein were cheeses (18%), processed meat (16%), domestic mammals (14%), and milk products (13%). The main sources of plant-based protein were bread (45%), pasta, rice, and other grains (7%), and nuts and seeds (7%). When comparing the current diet with the current PBMA scenario, the contribution of animal protein in the diet was reduced from 59% to 36%, with a considerable amount of animal protein still coming from cheese, dairy, fish, and eggs.

The soy, pea, wheat, and mycoprotein-based PBMA differed in protein quality. Soy and mycoprotein had a PDCAAS above 1.0, indicating these sources provide all the IAA in the required amounts, whereas pea and wheat had a PDCAAS of 0.73 and 0.34, respectively, indicating these sources lack 1 or more IAA in the required amounts. Mycoprotein had a digestibility of 78%, whereas soy, pea, and wheat had a digestibility of 92% or higher. The limiting amino acid of mycoprotein was leucine; for wheat, this was lysine; and for soy and pea, this was methionine + cysteine.

**FIGURE 2:**
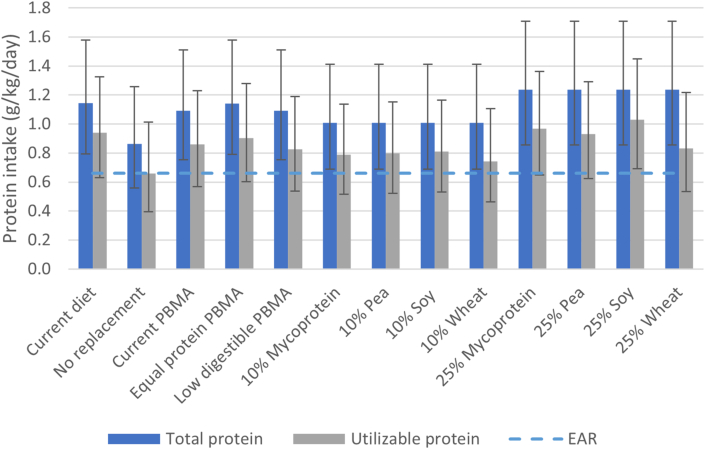
Median habitual total and utilizable protein intake (5th and 95th percentiles) in current diet and by scenario. EAR: estimated average requirement; PBMA: plant-based meat alternative.

For each scenario, the median habitual total and utilizable protein intakes are shown in Figure 2, and population adequacies in Table 3. Habitual total protein intake was 1.14 (0.79–1.58) g/kg/d in the current diet, with 99% of the population having an intake above the EAR of 0.66 g/kg/d. The no protein replacement scenario had the lowest habitual protein adequacy of 85%, and the 25% mycoprotein, pea, soy, and wheat scenarios had the highest adequacy of 100%.

**TABLE 3:** Population adequacy of habitual total and utilizable protein intake^1^ in current diet and by scenario.

Scenario	Total protein adequacy	Utilizable protein adequacy
	%	%
Current diet	99	93
No protein replacement	85	50
Current PBMA	99	86
Equal protein PBMA	99	90
Low digestible PBMA	99	81
10% Mycoprotein	97	76
10% Pea	97	78
10% Soy	97	79
10% Wheat	97	67
25% Mycoprotein	100	94
25% Pea	100	92
25% Soy	100	96
25% Wheat	100	81

Abbreviation: PBMA: plant-based meat alternative.

1 Protein adequacy: percentage of the population with habitual protein intake of >0.66 g/kg/d.

Habitual utilizable protein intake was 0.94 (0.63–1.32) g/kg/d in the current diet, with 93% of the population having utilizable protein intake above the EAR of 0.66 g/kg/d. The no protein replacement scenario had the lowest utilizable protein adequacy of 50%. The current PBMA scenario had a utilizable protein adequacy of 86%. Increasing the total protein content in the equal protein PBMA scenario raised adequacy to 90%, and reducing protein digestibility in the low digestible PBMA scenario lowered it to 81%. The 10% mycoprotein, pea, and soy scenarios resulted in utilizable protein adequacy varying from 76% to 79%, whereas the 25% mycoprotein, pea, and soy scenarios resulted in utilizable protein adequacies varying from 92% to 96% (Table 3). In contrast, the 10% and 25% wheat scenarios resulted in lower utilizable protein adequacies of respectively 67% and 81%.

For each scenario, the mean protein digestibility of the diet, total protein intake, PDCAAS, and the limiting amino acid per meal are shown in Supplemental Table 2. The mean protein digestibility was 88% in the current diet and ranged from 84% to 88% across the scenarios, with the 25% mycoprotein scenario representing the lowest diet digestibility. Lysine was the limiting amino acid across all scenarios at all meal occasions, except at dinner. Dinner in the current diet, current PBMA, equal protein PBMA, soy, and mycoprotein scenarios provided complete IAA, whereas dinner in the 10% and 25% wheat scenarios had the lowest PDCAAS of 0.78 and 0.71, respectively, with lysine being the limiting amino acid.

**FIGURE 3:**
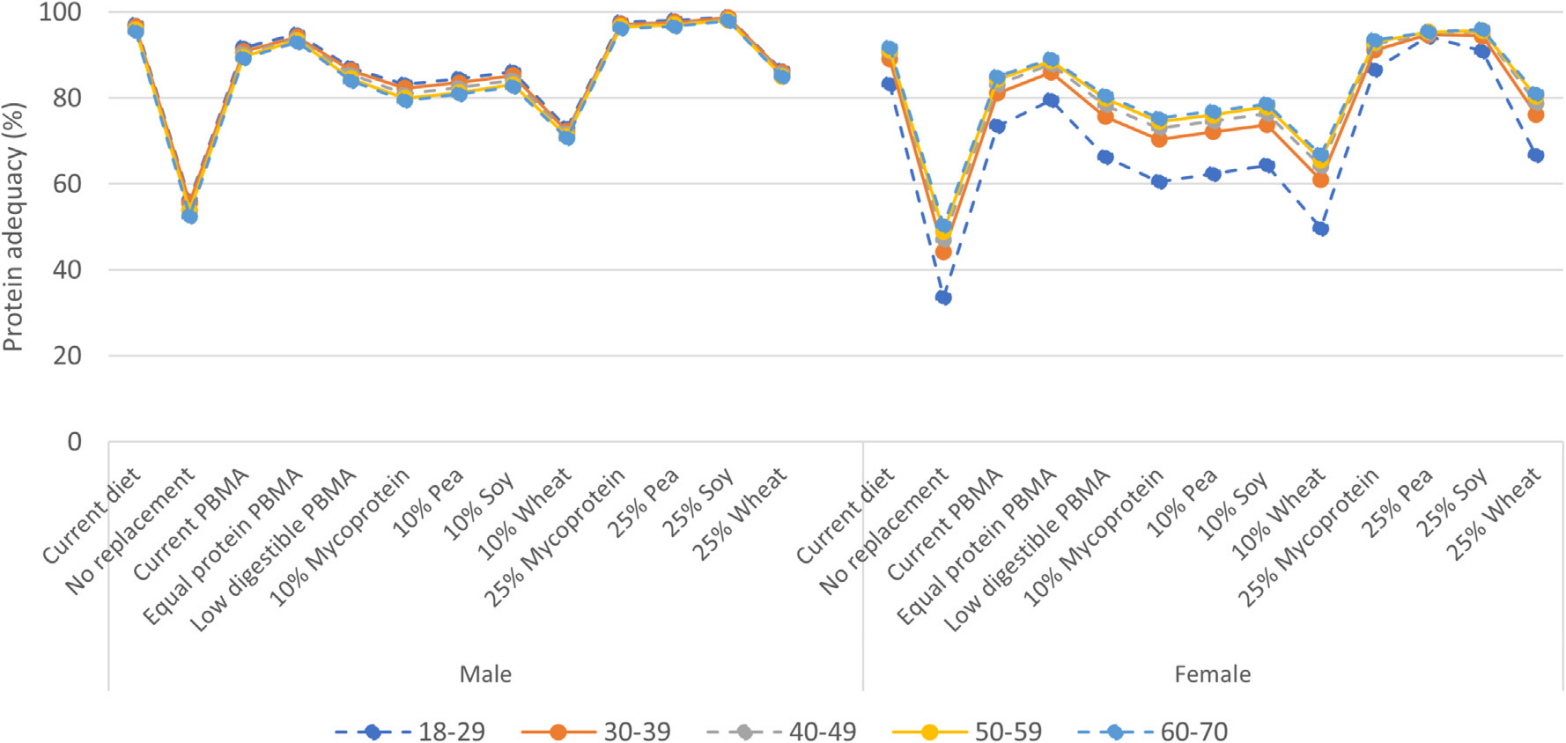
Population adequacy of habitual utilizable protein intake^1^ in current diet and by scenario by sex and age categories. (The lines are meant to recognize the pattern and they are not linear interpolations). PBMA, plant-based meat alternative. ^1^Protein adequacy: percentage of the population with habitual protein intake of >0.66 g/kg/d.

When stratifying the results by sex, in the current diet, females had a lower utilizable protein adequacy than males, respectively 89% and 96%. Across the scenarios, adequacy patterns were comparable between males and females. With increasing age, only small differences in intakes were observed, which were consistent across scenarios (Figure 3).

## Discussion

In this study, we modeled the impact of replacing animal meat with PBMA on protein intake and adequacy in the context of the current Dutch dietary pattern by taking into account protein quality. In the scenario where all animal meat was replaced with PBMA, utilizable protein adequacy decreased from 93% to 86% and remained adequate for the majority of the population. Increasing the total protein content in the current PBMA to that of animal meat raised adequacy from 86% to 90%, and reducing protein digestibility in the current PBMA to that of whole-food legumes lowered adequacy from 86% to 81%. Replacing high protein-high lysine PBMA with high protein-low lysine PBMA lowered adequacy from 92–96% to 81%. In the current diet, females had lower utilizable protein adequacy than males (89% compared with 96%), which was consistent across all scenarios and did not differ between age groups.

In this study, the protein adequacy of the diet was mostly impacted by the total protein content of the PBMA, but lysine content and protein digestibility of the PBMA also played a role. The scenarios where all animal meat was replaced with low lysine wheat-based meat resulted in the lowest protein adequacies, whereas replacements with higher lysine plant-based meat resulted in higher protein adequacies. In current diets, most plant protein comes from grains like wheat, which have low concentrations of lysine [10,28]. When replacing animal protein by adding more grain protein to the diet, there will be insufficient lysine to fulfill the amino acid requirements, and protein adequacy will decrease. Indeed, in the wheat scenarios and to a lesser extent in the current PBMA scenarios, grains became the primary source of protein in the diet (>49%) and, as a result, lowered protein adequacy. Together, the studied scenarios indicate that when plants are the primary source of protein, proteins should come from a variety of sources to ensure sufficient lysine.

Another factor impacting protein adequacy in the current study was protein digestibility. Compared to the current PBMA scenario, the low digestible PBMA scenario resulted in a lower protein adequacy. Interestingly, however, the scenario with the lowest digestible PBMA, being mycoprotein, did not result in the lowest protein adequacy. This finding can be explained by the high lysine content of mycoprotein compensating for the lower digestibility. Based on the current study, we hypothesize that the negative impact of a lower digestibility may be particularly relevant for foods that are lower in lysine. Processing such as milling, extracting, or cooking generally increases the protein digestibility of whole-food plant-based sources [9]. Recent research showed that the in vitro protein digestibility of soybeans and soy concentrate was 57% and 82%, whereas the digestibility of soy milk, tofu, and grilled soy-based meat was respectively 88%, 97%, and 91%[29,30]. Stable isotope studies in humans found that the ileal protein digestibility factor of pea protein was 94% [31], soy protein isolate was 92% [32], wheat protein isolate was 90% [33], cooked fava beans was 90% [34], cooked egg was 91% [35] and milk protein was 96% [36]. These studies suggest that ileal protein digestibility in humans is relatively high across a range of plant and animal-based foods. However, the ileal protein digestibility of PBMA remains to be established.

A factor that did not predict the protein adequacy of the diet was protein quality expressed as the PDCAAS of PBMA. Despite a difference in PDCAAS of 0.32 points between pea and soy-based PBMA, pea and soy scenarios resulted in comparable protein adequacy. The 10% pea and soy scenarios resulted in a protein adequacy of 78% and 79%, and the 25% pea and soy scenarios resulted in a protein adequacy of 92% and 96%. This indicates that protein quality overall, as expressed as PDCAAS of PBMA, did not predict the protein adequacy of the diet, whereas factors determining protein quality, being lysine content and protein digestibility, can be considered predictors.

Our findings are in agreement with earlier research on replacing animal protein with plant-based proteins. Simulation and observational data show that although in Western populations, the total protein intake in partially or fully plant-based diets is lower than in diets with animal-sourced foods, they generally meet country-specific protein requirements [37,38]. De Gavelle et al. [10] simulated a gradual substitution of animal protein foods with different mixtures of plant-based protein foods on protein adequacy. They found that with ≤50% of plant protein in the diet, protein, and amino acid intake was adequate with any mixture of plant-based proteins. With >50% plant protein, protein inadequacy was primarily determined by protein quantity, and only after >70% plant protein lysine became limiting, which lowered protein adequacy [10]. A recent randomized controlled trial concluded that a balanced vegan diet maintained daily muscle protein synthesis rates comparable to those of an isocaloric, isonitrogenous omnivorous diet in physically active, older adults [39], despite several short-term studies showing that consuming meat or omnivorous meals stimulated muscle protein synthesis more than PBMA or whole-food vegan meals [40,41],

In the current study, replacing all animal meat with current PBMA reduced the contribution of animal protein in the diet from 59% to 36%, with a considerable amount of animal protein still coming from dairy, fish, and eggs. Altogether, the findings suggest that when moving toward a diet with less animal protein, e.g., targeting ∼40% animal protein and ∼60% plant protein as currently proposed by expert groups and health authorities [4,42], average adult populations in Western countries will most likely still have sufficient quality protein intake.

In the current study, we observed that females had lower utilizable protein adequacy than males (89% compared with 96%), but we did not find lower adequacies with increasing age. This pattern was present in the current diet and was echoed over the replacement scenarios. This finding is in line with earlier population studies, which showed that with increasing age, absolute total protein intake reduces, but protein intake relative to reference body weight is stable [43]. However, protein requirements may increase with increasing age [44]. Together, this suggests that it should be ensured that individuals meet total protein intake requirements and consume proteins from a variety of sources, including cereals, legumes, nuts, and seeds. This recommendation also applies to people lowering animal protein from their diets, as this generally results in a lower total protein intake [38]. For this group, and in particular, in places where there is not a strong tradition of vegetarian or vegan diets, it is unsure how the transition toward more plant-based diets will happen in practice. This transition could mean a move to plant-based foods that have little protein, potentially increasing the risk of not getting enough total protein [11]. For example, the protein content per 100 g of chickpeas is 7.6 g, lentils is 8.8 g, wholegrain bread is 11.1 g, tofu is 12.4 g, and plant-based meat is 15.4 g [15]. Individuals may benefit from adding PBMA to their diet because these products are among the plant-based foods that are highest in protein.

In the present study, we only looked at the impact of protein from PBMA and not the impact of other nutrients. Earlier simulation studies replacing animal meat and dairy with plant-based foods showed improvements in the nutritional quality but also some potential nutrient inadequacies [[45], [46], [47]]. Studies focusing on PBMA indicated that replacing animal meat with PBMA improved fiber and saturated fat intake but resulted in inadequate concentrations of vitamin B12, vitamin B2, and bioavailable zinc and iron [47]. The simulation studies also indicated that inadequate levels can potentially be compensated for by fortification [46]. To ensure PBMA-based diets fit in a healthy diet, they should be formulated with minimal salt and saturated fat and fortified with key nutrients for which there is a risk of deficiency if animal products are replaced [42]. However, individuals consuming PBMA may also make other dietary choices beyond simply replacing animal meat with PBMA, which can impact overall dietary intake. Due to the lack of data on this topic, it is crucial to study the diet quality of PBMA consumers in real life to inform the development of dietary recommendations.

A strength of this study is that it is 1 of the first studies exploring the impact of consuming a representative sample of novel protein-rich plant-based foods on protein intake and adequacy although considering protein quality. The wide range of scenarios allowed the exploration of factors influencing protein adequacy. Additionally, we applied advanced methods to calculate habitual utilizable protein intake by meal moment [15]. Given that there is a lack of scientific consensus on whether to evaluate utilizable protein based on meal moments rather than total daily intake [11,12], our study adopted the meal moment-based approach as the most restrictive option. To provide population-representative intakes, we calculated habitual intakes by correcting daily protein intakes for the intra-individual (day-to-day) variation and weighting for demographics, seasons, and week or weekend days using SPADE [26]. Finally, we calculated individual protein requirements by using reference body weight; this accounts for the increase in overweight in the population [25] and brings protein requirement based on body weight closer to protein requirement based on fat-free mass [48].

A limitation of this study is that we measured protein quality with PDCAAS instead of digestible IAA score (DIAAS), which is the preferred method to evaluate protein quality [22]. At present, ileal digestibility data for single amino acids, as required DIAAS, are not yet available for the wide range of foods needed for a modeling study. To account for the limitation of PDCAAS that it often overestimates digestibility [22], we, on purpose, selected a conservative estimate of digestibility in the low-digestibility scenario. Additionally, it should be noted that this study was done in the Dutch population, which has a relatively high protein intake from animal meat and dairy sources. After replacing all meat, a considerable amount of protein still originated from dairy, fish, and eggs. Although this dietary pattern is representative of Western European dietary patterns, the results of this modeling study may not be representative of populations with lower animal protein intakes. It would be beneficial to repeat this study in such populations. Finally, in the theoretical replacement scenarios, we deliberately made a strict assumption by evaluating utilizable protein based on meal moments rather than total daily intake, and we omitted some real-life factors, such as partial replacement of animal meat intake (representing flexitarian diets) and using protein blends (representing real-life products). As a result, in the scenario where all animal meat was replaced with PBMA, utilizable protein adequacy decreased from 93% to 86%. However, in real-life situations such as flexitarian diets that include PBMA from blended protein sources, utilizable protein adequacy would likely be somewhere between 86% and 93%.

In conclusion, this research in the context of a Western European diet indicated that in an extreme scenario where all animal meat is replaced with PBMA, utilizable protein remained adequate for the majority of the population, with a slight decrease from 93% to 86%. Protein adequacy was mostly impacted by total protein content, lysine content, and protein digestibility of the PBMA. For people who get the majority of their protein from plant sources, a primary focus should be to ensure that a sufficient total protein content comes from a variety of sources.

## Author contributions

The authors’ responsibilities were as follows– AJW, SNH, SB, MD-K: designed research; AJW, SNH: conducted research; AJW: analyzed data; AJW, SNH, SB, MD-K: wrote the paper; AJW: had primary responsibility for final content; and all authors: read and approved the final manuscript.

## Data availability

Data from the Dutch National Food Consumption Survey 2012–2016 can be requested at https:// www.rivm.nl/en/dutchnational-food-consumption-survey/data-on-request, and the Dutch Food Composition Database can be accessed at https://www.rivm.nl/en/dutch-food-composition-database.

## Funding

This study was funded by Unilever Foods Innovation Centre, Wageningen, The Netherlands.

## Conflict of interest

AJW and MD-K are employees of Unilever, a global company that produces and markets a variety of foods and beverages, including plant-based meat alternatives. All other authors report no conflicts of interest.
