# Supplementary material for: Replacing Animal Meat with Plant-Based Meat Alternatives: The Impact of Protein Quality on Protein Adequacy in the Dutch Diet
Source: Curr Dev Nutr. 2025 Feb 5;9(3):104562. doi: 10.1016/j.cdnut.2025.104562 (PMC11928959; doi:10.1016/j.cdnut.2025.104562)
Supplement: Multimedia component 1 [file mmc1.docx]

**Replacing animal meat with plant-based meat alternatives: the impact of protein quality on protein adequacy in the Dutch diet**

Anne J. Wanders^1^*, Samantha N. Heerschop^2^, Sander Biesbroek^2^, Mariska Dötsch-Klerk^1^

^1^ Unilever Foods Innovation Centre, Wageningen, The Netherlands

^2^ Division of Human Nutrition and Health, Wageningen University and Research, Wageningen, The Netherlands

*Corresponding author. Email: [anne.wanders@unilever.com](mailto:anne.wanders@unilever.com)

**Index of Supplemental Data**

- Supplemental Text 1
- Supplemental Table 1: Protein sources, amino acid composition and contribution of each source to current PBMA
- Supplemental Table 2: Total diet protein digestibility, PDCAAS and limiting amino acid in current diet and by scenario

**Supplemental Text 1**

In the EU any ingredients that appear in the name of the product, are emphasised on the label in words, pictures or graphics, or are essential to characterise the food need to be quantified in percentages in the ingredient list ([Food labelling - general EU rules - Your Europe](https://europa.eu/youreurope/business/product-requirements/food-labelling/general-rules/index_en.htm)).

In the current evaluation, in half of the products protein came from a single ingredient (100%). In other products protein blends were reported on ingredient lists. Here we show a translated example of an ingredient list:

**Ingredients:** rehydrated SOY proteins 87.0% (water, SOY proteins 33.0%), rapeseed oil, rehydrated WHEAT proteins 2.5% (water, WHEAT proteins 1.0%), maltodextrin, alcohol vinegar, spices (onion, garlic, cumin, black pepper, coriander, nutmeg, allspice, turmeric, paprika), yeast extract, BARLEY malt extract, salt, flavoring, mineral salt (potassium chloride), caramelized sugar, acidity regulator (potassium hydroxide).

In this particular product we extracted 87g soy protein and 2.5g wheat protein per 100g product, resulting in 97% of total protein originating from soy.

Of the 121 PBMAs, five ingredients lists did not contain percentages, these were imputed based on matching ingredient lists.

**Supplemental Table 1: Protein sources, amino acid composition and contribution of each source to current PBMA**

| **Protein sources ^1^** | **Contribution** | **digestibility** | **His** | **Ile** | **Leu** | **Lys** | **Met+ Cys** | **Phe+ Tyr** | **Thr** | **Trp** | **Val** | **PDCAAS^2^** |
| --- | --- | --- | --- | --- | --- | --- | --- | --- | --- | --- | --- | --- |
|  | % |  | AA composition (mg/g protein)^1^ | | | | | | | | |  |
| Soy | 55 | 95 | 26 | 45 | 77 | 65 | 26 | 87 | 41 | 14 | 48 | 105 |
| Wheat | 23 | 95 | 20 | 34 | 67 | 17 | 31 | 84 | 26 | 10 | 38 | 34 |
| Pea | 11 | 92 | 25 | 44 | 80 | 71 | 18 | 90 | 37 | 10 | 50 | 73 |
| Milk | 5 | 95 | 29 | 58 | 96 | 90 | 33 | 93 | 43 | 14 | 69 | 136 |
| Fava | 4 | 86 | 28 | 33 | 74 | 60 | 17 | 65 | 31 | 6 | 39 | 64 |
| Mycoprotein | 3 | 78 | 25 | 49 | 79 | 81 | 37 | 86 | 53 | 17 | 61 | 101 |
| Current PBMA | 100 | 94 | 25 | 43 | 76 | 56 | 26 | 86 | 37 | 12 | 47 | 108 |

Abbreviations: AA: amino acid; Cys: cysteine; His: histidine; Ile: isoleucine; Leu: leucine; Lys: lysine; Met: methionine; PBMA: plant-based meat alternative; PDCAAS: protein digestibility-corrected amino acid score; Phe: phenylalanine; Thr: threonine; Trp: tryptophan; Tyr: tyrosin; Val: valine.

^1^ Amino acid composition data on soy, pea, wheat, mycoprotein taken from [1], on fava bean (cooked) from [2], and on milk from [3].

^2^ Older child, adolescent, and adult adults reference pattern from [4]

**Supplemental Table 2: Mean total diet protein digestibility and total protein intake, PDCAAS and limiting amino acid per meal moment in current diet and by scenario**

| **Meal moment**  **(total protein intake) ^1^** | Protein digestibility | **Before breakfast**  **(0.7)** | | **Breakfast**  **(13.1)** | | **Morning snack**  **(3.8)** | | **Lunch**  **(22.0)** | | **Afternoon snack**  **(4.7)** | | **Dinner**  **(31.8)** | | **Evening snack**  **(6.5)** | |
| --- | --- | --- | --- | --- | --- | --- | --- | --- | --- | --- | --- | --- | --- | --- | --- |
|  | % | PDCAAS | LAA | PDCAAS | LAA | PDCAAS | LAA | PDCAAS | LAA | PDCAAS | LAA | PDCAAS | LAA | PDCAAS | LAA |
| Current diet | 88 | 0.05 | Met+Cys | 0.94 | Lys | 0.38 | Lys | 1.03 | Lys | 0.44 | Lys | 1.10 | (Leu) | 0.49 | Lys |
| No protein replacement | 86 | 0.05 | Met+Cys | 0.89 | Lys | 0.36 | Lys | 0.88 | Lys | 0.41 | Lys | 0.93 | Leu | 0.46 | Lys |
| Current PBMA | 88 | 0.05 | Met+Cys | 0.91 | Lys | 0.37 | Lys | 0.93 | Lys | 0.42 | Lys | 1.03 | (Lys) | 0.48 | Lys |
| Equal protein PBMA | 88 | 0.05 | Met+Cys | 0.92 | Lys | 0.37 | Lys | 0.94 | Lys | 0.42 | Lys | 1.04 | (Lys) | 0.48 | Lys |
| Low digestible PBMA | 85 | 0.05 | Met+Cys | 0.90 | Lys | 0.37 | Lys | 0.90 | Lys | 0.42 | Lys | 0.95 | Lys | 0.47 | Lys |
| 10% Mycoprotein | 85 | 0.05 | Met+Cys | 0.91 | Lys | 0.37 | Lys | 0.94 | Lys | 0.43 | Lys | 0.99 | Leu | 0.48 | Lys |
| 10% Pea | 87 | 0.05 | Met+Cys | 0.92 | Lys | 0.37 | Lys | 0.95 | Lys | 0.43 | Lys | 0.98 | Met+Cys | 0.48 | Lys |
| 10% Soy | 88 | 0.05 | Met+Cys | 0.91 | Lys | 0.37 | Lys | 0.94 | Lys | 0.43 | Lys | 1.05 | (Leu) | 0.48 | Lys |
| 10% Wheat | 88 | 0.05 | Met+Cys | 0.88 | Lys | 0.36 | Lys | 0.85 | Lys | 0.41 | Lys | 0.78 | Lys | 0.46 | Lys |
| 25% Mycoprotein | 84 | 0.05 | Met+Cys | 0.93 | Lys | 0.37 | Lys | 0.98 | Lys | 0.43 | Lys | 1.01 | (Leu) | 0.48 | Lys |
| 25% Pea | 88 | 0.05 | Met+Cys | 0.93 | Lys | 0.37 | Lys | 0.99 | Lys | 0.43 | Lys | 0.95 | Met+Cys | 0.48 | Lys |
| 25% Soy | 89 | 0.05 | Met+Cys | 0.93 | Lys | 0.37 | Lys | 0.98 | Lys | 0.43 | Lys | 1.09 | (Leu) | 0.48 | Lys |
| 25% Wheat | 89 | 0.05 | Met+Cys | 0.88 | Lys | 0.36 | Lys | 0.82 | Lys | 0.41 | Lys | 0.71 | Lys | 0.46 | Lys |

Abbreviations: Cys: cysteine; LAA: limiting amino acid; Leu: leucine; Lys: lysine; Met: methionine; PBMA: plant-based meat alternative; PDCAAS: protein digestibility-corrected amino acid score.

^1^ Average total protein intake (g/day) in Current Diet

**References**

1. Švarc PL, Jensen MB, Langwagen M, Poulsen A, Trolle E, and Jakobsen J (2022) Nutrient content in plant-based protein products intended for food composition databases. J Food Compos Anal. 106:104332. DOI: 10.1016/j.jfca.2021.104332

2. Nosworthy MG, Medina G, Franczyk AJ, Neufeld J, Appah P, Utioh A, Frohlich P, and House JD (2018) Effect of Processing on the In Vitro and In Vivo Protein Quality of Beans (Phaseolus vulgaris and Vicia Faba). Nutrients. 10:671. DOI: 10.3390/nu10060671

3. Heerschop SN, Kanellopoulos A, Biesbroek S, and van 't Veer P (2023) Shifting towards optimized healthy and sustainable Dutch diets: impact on protein quality. Eur J Nutr. 62:2115-2128. DOI: 10.1007/s00394-023-03135-7

4. Food and Agriculture Organization of the United Nations (2013) Dietary Protein Quality Evaluation in Human Nutrition: Report of an FAO Expert Consultation, 31 March-2 April, 2011, Auckland, New Zealand. Food and Agriculture Organization of the United Nations, Rome
